# Intimate partner violence and early child growth: a community-based cohort study in Nicaragua

**DOI:** 10.1186/1471-2431-12-82

**Published:** 2012-06-22

**Authors:** Mariano Salazar, Ulf Högberg, Eliette Valladares, Lars-Åke Persson

**Affiliations:** 1 Center for Demography and Health Research, Nicaraguan National Autonomous University, León, Nicaragua; 2 Epidemiology and Global Health, Department of Public Health and Clinical Medicine, Umeå University, Umeå, Sweden; 3 Department of Women's and Children's Health, Uppsala University, Uppsala, Sweden; 4 Obstetrics and Gynecology, Department of Clinical Sciences Umeå University, Umeå, Sweden; 5 Department of Obstetrics and Gynecology, Nicaraguan National Autonomous University, León, Nicaragua; 6 International Maternal and Child Health (IMCH), Department of Women’s and Children’s Health, Uppsala University, Uppsala, Sweden

**Keywords:** Intimate partner violence, Child growth, Gender

## Abstract

**Background:**

This study analyses whether a mother’s exposure to different forms of Intimate Partner Violence (IPV) during pregnancy was associated with the index child linear growth, and whether these associations were modified by the gender of the child.

**Methods:**

A pregnancy cohort of 478 women in León, Nicaragua, resulted in 461 live births. From this group, 81% (375/461) children were available for anthropometric follow-up at 40 to 46 months. Analysis of covariance (ANCOVA) was used to assess the association between IPV and height-for-age Z-scores, adjusting for confounding factors.

**Results:**

Sixty-three percent (236/375) of the mothers had been exposed to some form of IPV during pregnancy (emotional, physical, sexual or controlling behavior). After adjustment for confounding factors, maternal exposure to any IPV during pregnancy was associated with 0.24 lower mean height-for-age Z-scores (p = 0.02). A separate analysis of each IPV type showed that emotional, physical or sexual IPV during pregnancy were not significantly associated with lower mean height-for-age Z-scores, whereas ever exposure to controlling behavior by the father of the child was related to 0.29 lower mean height-for-age Z-scores (p < 0.01) When stratified by gender, these associations remained significant only for young girls.

**Conclusions:**

This study has contributed to the growing amount of evidence pointing to the pervasive effect of different forms of IPV on child health. Our study highlights the relevance of maternal autonomy for linear child growth, especially for young girls in the Nicaraguan context.

## Background

Intimate Partner Violence (IPV) is a widespread public health problem that affects millions of women worldwide [1] and has severe physical and mental health consequences for mothers [2] and their children [3,4]. Women who experience physical or sexual IPV are often also exposed to a high level of controlling behavior by their partners [1].

Impaired growth is an important health determinant in children and has been associated with higher morbidity and mortality in children under 5 years [5]. Different forms of Violence against Women (VAW) have been identified as factors related to impaired child growth in diverse cultural settings especially in low income countries. For example, one Indian cross-sectional community-based study, with children aged 12–35 months, reported that women’s exposure to physical abuse by any family member in the previous twelve months was associated with wasting, stunting, and severe underweight [6]. Rico et al. using cross-sectional national data from five demographic and health surveys in Africa and Latin America, found a positive association between maternal exposure to physical IPV and child stunting in one of the settings (Kenya) and a positive non-significant trend in the others [7]. Further, a hospital-based case–control study in Brazil found that severe physical partner abuse in the previous twelve months increased the risk of severe acute malnutrition fourfold in children aged 1 to 24 months [8].

Few longitudinal studies have analyzed the association between VAW and child growth. A recent community-based cohort study in Bangladesh found that exposure to any form of family violence was associated with lower weight-for-age, height-for-age, and weight-for-height Z-scores at birth and at 24 months, especially in boys [3]. However, the effect IPV has on child growth might be different in high income countries. Two recent prospective studies conducted in the United States support this hypothesis. These studies found that maternal exposure to IPV was associated with higher odds of obesity during childhood and adolescence [9,10].

### Conceptual framework

Child growth and survival can be determined by an intersection between social, economical, individual and environmental factors [11,12]. These factors can interact in several ways creating different pathways affecting the child’s and the child’s primary caregiver (mother) health which in turn will influence the child growth. VAW is a key factor that has been repeatedly associated with impaired maternal and child health [2,3,13,14].

Yount et al. in a landmark review article analyzing evidence from 31 studies around the world, have described the different pathways by which VAW can impair early child growth [12]. For example, maternal exposure to VAW can increase women´s prenatal risk behaviors and diminish their access to prenatal care, which can have a strong effect on the fetus growth and pregnancy outcomes [12]. In addition, maternal increased stress during pregnancy can deregulate the fetus stress-response system. On the other hand, VAW can also interfere with postnatal child care, decreasing the likelihood that the child will receive adequate preventive care or therapeutic treatment when sick [12]. The gender of the child has been identified by Krantz and García-Moreno and Yount et al. as another important determinant of children´s exposure to inadequate care and poor growth [12,15]. In many cultural settings there is a clear difference in the way young boys and girls are taken care of, with young girls more prone to receive inadequate care than boys [15].

### Rationale

Five out of ten ever married Nicaraguan women have experienced physical IPV during their lifetime [16], and one in ten during pregnancy [17]. In addition, exposure to IPV in this setting has been related to higher risks of low birthweight [18] and under-five mortality [19]. However, to the best of our knowledge, there are no longitudinal studies in Latin America analyzing the association between mother’s exposure to IPV and child linear growth. Thus, the aim of this cohort study is to analyze whether there is an association between exposure to IPV during pregnancy and linear growth of the child, adjusting for relevant confounding factors. The study will further examine if the gender of the child modifies such an association between violence and child growth.

## Methods

### Study setting and procedures

The study was conducted in a health and demographic surveillance system (HDSS) covering 22% (n = 54,647 people) of the urban and rural population of León municipality, Nicaragua [20]. The HDSS is a dynamic cohort that provides yearly information on births, deaths, immigration, and emigration, allowing longitudinal follow-up of study participants.

The starting point of this research is a population-based survey assessing the magnitude and characteristics of IPV during pregnancy [17]. In this study, all pregnant women living in the areas covered by the HDSS (n = 483) were identified by a screening question posted in the HDSS baseline data collection and then invited to participate. One woman refused to join the survey and two were found not to be pregnant. During data collection we identified that two households had a pair of pregnant women living together, due to the sensibility of the study only one woman in each household was randomly selected to be interviewed. Finally, 478 women were included. This paper includes a median follow-up time of 43 months (IQR 40–45 months) of all children born alive. Women were enrolled between 2002 and 2003.

### Data collection

A modified version of the WHO Multi-country Study on Women’s Health and Domestic Violence against Women questionnaire [1] was used to measure exposure to IPV. The WHO instrument was chosen because it had been extensively used to measure IPV magnitude in developing countries and the items within each IPV sub-scale have shown high internal consistency [21]. The WHO questionnaire was modified by including questions about emotional, physical or sexual IPV during pregnancy**.**

The questionnaire contains four sub-scales measuring different IPV types (emotional, physical, sexual and controlling behavior). Each sub-scale has specific questions depicting violent actions that a man can do to his female partner. Yelling, humiliation, intimidation, and threats were considered emotional IPV. Exposure to any violent partner acts such as slaps, pushes, punches, kicks, strangulation, and use of weapons were labeled as physical IPV. The partner use of force/threats or intimidation to have sexual relations with the woman was typified as sexual IPV. A woman was classified as experiencing IPV if she answered “yes” to any of the questions explored within each sub-scale.

Exposure to controlling behavior by the father of the child was assessed on a scale containing seven items describing the following actions: if the partner restricted the woman’s contacts with friends; if the partner restricted the woman’s contacts with family; if he insisted on knowing her whereabouts at all times; if he ignored and treated her indifferently; if he became angry if she spoke to another man; if he was constantly suspicious that she was unfaithful; and if he expected her to ask for his permission to seek healthcare for herself. These were later summarized into two categories: no controlling behaviors or exposed to 1–7 controlling behaviors.

Emotional, physical and sexual IPV were assessed if they occurred in a lifetime or during pregnancy. In order to enhance disclosure, data on emotional, physical and sexual IPV were collected twice during pregnancy. The average gestational age at the first visit was 19 weeks and 36 weeks at the second. Only lifetime exposure to controlling behavior by father of the child was collected.

In our study all IPV sub-scales showed high internal consistency with Cronbach alphas above 0.80 for emotional (0.83), physical (0.89), sexual (0.82) and controlling behavior by the father of the child (0.81) sub-scales. We also performed exploratory factor analysis taking into account the questions related to IPV (controlling behavior, emotional, physical and sexual violence). The factor analysis showed four factors with eigen values greater than one. A Varimax rotation was applied to these factors because they were considered to be independent. The first factor extracted represented questions exploring physical IPV, the second factor represented questions related to controlling behavior, the third factor represented questions related to sexual IPV and the fourth factor questions related to emotional IPV (data not shown). The four factors had a cumulative variance of 0.6271 . The analysis showed that the WHO instrument can distinguish between different forms of IPV in the Nicaraguan setting.

Data on birthweight were excerpted from hospital and clinic records. At a median child age of 43 months, standing height was measured using a vertical metric rule attached to the scale and a horizontal headboard. The metric rule was graduated at 0.1 cm intervals. During the visit, two standing height measurements were collected and their mean calculated.

Demographic data (women’s age, marital status, residency, education, parity, and socioeconomic status) were collected during pregnancy. Socioeconomic status was measured by means of the Unsatisfied Basic Needs Assessment index [22]. This variable was constructed from four indicators: inadequate housing (if the family home had a dirt floor or walls constructed from materials other than cement), low school enrolment (if there was a child of school age in the household not in education), high dependency economy (if the ratio between non-working and working persons in the household was higher than one) and inadequate sanitary conditions (if no piped water was available indoors or there was no flush toilet). Households with two or more such indicators were considered poor.

Emotional distress during pregnancy was measured using the Self-Report Questionnaire (SRQ), a 20-item questionnaire exploring depressive and neurotic symptoms that has been validated in Nicaragua [23] . Distress was defined as a score ≥7 [23].

### Data handling and quality control

Two female interviewers collected the data and performed the anthropometric measurements. They received extensive theoretical and practical training on how to measure and record height following WHO standardized procedures [24]. The equipment was checked before each day of use. A field supervisor was always present during the anthropometric measure to ensure the quality of the data. The data were stored in a database created in Access 2000. It contained validation rules to avoid typing errors. Once the database was completed, the data were cleaned to ensure quality.

### Data analysis

Z-scores are the preferred way to express anthropometric indices because they are used to calculate summary statistics [24]. The program ANTHRO 2005 (WHO, 2006) was used to convert mean height follow-up measurements into height-for-age Z-scores. Height-for-age index was used to evaluate cumulative linear growth; low values were associated with impaired long-term growth.

Student’s *t*-test was used to compare differences in the mean height-for-age Z-scores values for the mother’s residency, education, parity, socioeconomic status, maternal emotional distress and child gender. Birthweight, child age, mother’s age at pregnancy were not normally distributed, thus Spearman correlation was used to assess the relationship between these variables and height-for-age Z-scores. Mann–Whitney *U* test was used to compare median mothers’ age across IPV groups (data not shown).

Analysis of covariance (ANCOVA) was used to assess the association between different types of IPV and height-for-age Z-scores adjusted for confounding factors. Height-for-age was normally distributed (Kolmogorov-Smirnov test p value > 0.05) and the variance was homogeneous among all groups (Levene’s test p value > 0.05). Mother’s educational level, age, residency, parity, socioeconomic status, emotional distress during, child age and birthweight were considered potential confounders. A variable was included in the model if it had a p value <0.20 in its association with IPV exposure and height-for-age Z-scores and if there was a difference of 5% or more between the crude and adjusted estimates. Birthweight could or could not be considerate an intermediate factor between exposure to IPV during pregnancy and subsequent growth during early childhood. To test this hypothesis, in preliminary analysis we included birthweight as a confounding factor, however the difference between crude and adjusted estimates was less than 1% (data not shown). Thus, birthweight was excluded of the final analysis. The confounding factors included in each final model are described in the footnotes of Table 1.

**Table 1:** Adjusted mean height-for-age Z-scores and 95% CI by exposure to different forms of Intimate Partner Violence (IPV) during pregnancy

**IPV exposure**		**Adjusted mean height-for-age Z-scores (95% CI)**		
		**Boys n = 178**	**Girls n = 197**	**Total n = 375**
Any IPV during pregnancy † ‡	Yes	−1.03 (−1.20 to −0.87)	−1.14 (−1.32 to −0.96)	−1.09 (−1.21 to −0.98)
	No	−0.96 (−1.18 to −0.73)	−0.76 (−0.98 to −0.54)*	−0.85 (−1.01 to −0.69)*
Emotional IPV during pregnancy ‡	Yes	−0.92 (−1.15 to −0.68)	−1.05 (−1.31 to −0.79)	−0.99(−1.16 to −0.81)
	No	−1.05 (−1.21 to −0.89)	−0.97 (−1.14 to −0.80)	−1.01 (−1.12 to −0.89)
Physical IPV during pregnancy §	Yes	−1.07 (−1.43 to −0.71)	−1.20 (−1.58 to −0.81)	−1.13 (−1.40 to −0.87)
	No	−1.00 (−1.14 to −0.85)	−0.96 (−1.11 to −0.81)	−0.98 (−1.08 to −0.88)
Sexual IPV during pregnancy ∥	Yes	−1.02 (−1.53 to −0.51)	−1.04 (−1.63 to −0.44)	−1.03 (−1.42 to −0.64)
	No	−1.01 (−1.15 to −0.86)	−0.99 (−1.14 to −0.84)	−1.00 (−1.10 to −0.89)
Controlling behavior by partner ‡	Yes	−1.03 (−1.20 to −0.85)	−1.23 (−1.42 to −1.03)	−1.13 (−1.26 to −1.00)
	No	−0.98 (−1.19 to −0.77)	−0.74 (−0.94 to −0.54)*	−0.84 (−0.99 to −0.70)*

* p < 0.05. † Emotional, physical, or sexual violence or controlling behavior by partner. ‡ Adjusted for residency (urban or rural), mother’s education (≤ 3^rd^ grade, ≥ 3^rd^ grade), and child age (months). § Adjusted for mother’s education (≤ 3^rd^ grade, ≥ 3^rd^ grade) and residency (1 and ≥2). ∥ Adjusted for parity (1 and ≥2).

In addition, multivariate analyses were stratified by the child gender. The Statistical Package for Social Sciences (Version 15; SPSS Inc., Chicago, IL) was used to analyze the data. All bivariate and multivariate analysis were considered significant if p < 0.05.

### Ethics

The study protocol was approved by the Ethics Research Committee of León University, and WHO ethical guidelines for research on domestic violence were followed [25]. Written informed consent was obtained from the mothers prior to data collection. To ensure confidentiality, names were removed from the database. Further, the female interviewers received extensive training to interview women in privacy, with sensitivity, empathy, and without expressing judgment. Weekly debriefing was conducted with the interviewers to relieve stress. All mothers participating in the study were given a leaflet with information about institutions providing free health and legal services in their community. In addition, frequent meetings with a trained psychologist allowed the interviewers to relieve stress.

## Results

A pregnancy cohort of 478 women was followed, resulting in 461 live births. Of these, 81% children (375/461) were available for anthropometric assessment, a median of 43 months (IQR 40 to 45 months) after birth. Of the children lost to follow-up, 78 migrated and eight died from different causes during their first year of life. No significant difference in residency, education, parity, and IPV exposures was found between included children and those lost to follow-up (data not shown).

### Characteristics of the mothers and exposure to IPV

The median age at pregnancy was 23 years (interquartile range 19 to 27 years). Seven out of ten women lived in an urban area (256/375) and half had more than three years of education (208/375). Sixty one percent (230/375) had more than one child, and 57% (215/375) were classified as poor. Thirty nine percent (146/375) reported high emotional distress during pregnancy (Table 2).

**Table 2:** Relationship of the mother’s characteristics and the children´s gender to the children’s height-for-age Z-scores, n = 375

**Characteristics**	**n (%)**	**Height-for-age Z-scores**		
		**Mean**	**SD**	P value*
Place of residency				
Rural	119 (32)	−1.27	0.87	
Urban	256 (68)	−0.87	1.01	<0.01
Mother’s education				
≤ 3rd grade	167 (45)	−1.26	0.96	
> 3rd grade	208 (55)	−0.79	0.95	<0.01
Parity				
≥ 2	230 (61)	−1.15	0.97	
1	145 (39)	−0.77	0.96	<0.01
S. status				
Poor	215 (57)	−1.14	0.90	
Non-poor	160 (43)	−0.81	1.01	<0.01
Emotional distress				
SRQ ≥ 7	146 (39)	−1.21	0.93	
SRQ ≤ 6	229 (61)	−0.87	0.99	<0.01
Child gender				
Boy	178 (47)	−1.01	0.93	
Girl	197 (53)	−0.99	1.03	0.90

*Student’s *t*-test, SD (standard deviation).

IPV exposure was common. Overall, 73% (275/375) of the mothers experienced any lifetime IPV (emotional, physical, sexual, or controlling behavior) and 63% (236/375) reported any violence during the current pregnancy (Table 3).

**Table 3:** Exposure to Intimate Partner Violence (IPV) among Nicaraguan mothers in the study

**Type of violence**	**% of all mothers, n = 375**
Any lifetime violence*	73
Lifetime emotional violence	54
Lifetime physical violence	31
Lifetime sexual violence	14
Any violence during pregnancy†	63
Emotional violence during pregnancy	32
Physical violence during pregnancy	13
Sexual violence during pregnancy	7
Controlling behavior by father of the child	55

*Ever exposure to emotional, physical, or sexual IPV or controlling behavior by the father of the child. †Exposure during pregnancy to emotional, physical, or sexual IPV or controlling behavior by the father of the child.

### Association between IPV and women’s characteristics

Bivariate analysis showed few significant differences between women exposed or not to IPV during pregnancy. Median mothers’ age was lower among women exposed to any form of IPV during pregnancy (p < 0.05). Mothers living in a rural area were more often ever exposed to controlling behavior by the father of the child (p < 0.05). Any IPV during pregnancy, emotional violence during pregnancy, and ever controlling behavior by the father of the child were more common among women with three years or less of education (p < 0.05). Emotional distress was higher among women exposed to any form of IPV during pregnancy (p < 0.05). Parity and socioeconomic status were not significantly associated with any form of IPV during pregnancy (p > 0.05) (data not shown).

### Participating children

Fifty-three percent (197/375) of the participating children were girls. The median birthweight of the children was 3100 g (interquartile range 2880 to 3400 g). Two percent (9/375) were of low birthweight (< 2500 g). The median age at anthropometric examination was 43 months (interquartile range 40 to 45 months). At follow up, mean height-for-age Z-score was −1.00 ± 0.98 SD, ranging from −4.45 to 2.89.

### Bivariate association between child and mother’s characteristics and height-for-age Z-scores

Child age and birthweight were positively correlated with height-for-age Z-scores (p < 0.01), however maternal age was not (p = 0.05) (Table 4). The mothers’ low educational level, rural residency, multiparity, poor socioeconomic status and high emotional distress during pregnancy were associated with lower mean height-for-age Z-scores (p < 0.05) (Table 2). Mean height-for-age Z-scores did not significantly differ by the gender of the child (p > 0.05) (Table 2).

**Table 4:** Spearman correlation coefficient between height-for-age Z-scores, mother’s age at pregnancy, child age at follow-up and birthweight, n = 375

**Variable**	**Median** **(Interquartile range)**	**Spearman correlation coefficient (p value)**
Mother age (years)	23 (19 to 27)	0.10 (p = 0.05)
Child age (months)	43 (40 to 45)	0.17 (p < 0.01)
Birthweight (g)	3100 (2880 to 3400)	0.24 (p < 0.01)

### Association between IPV exposure and child growth

Adjusted mean height-for-age Z-scores are shown on Table 1. After adjustment for confounding factors, maternal exposure to any IPV during pregnancy (emotional, physical, sexual or ever controlling behavior by the father of the child) was associated with 0.24 lower mean height-for-age Z-scores (p = 0.02). A separate analysis of each IPV type showed that emotional, physical or sexual IPV during pregnancy were not significantly associated with lower mean height-for-age Z-scores, whereas ever exposure to controlling behavior by the father of the child was related to 0.29 lower mean height-for-age Z-scores (p < 0.01) (Table 1).

Gender differences were also found. Exposure to any IPV during pregnancy and to ever controlling behavior by the father of the child associated with lower height-for-age Z-scores among girls but not among boys. Girls whose mothers had been exposed to any IPV during pregnancy had 0.38 lower mean height-for-age Z-scores than those not exposed (p < 0.01). In addition, girls whose mothers had been ever exposed to controlling behavior by the father of the child had 0.49 lower mean height-for-age Z-scores than those not exposed (p < 0.01) (Table 1).

## Discussion

This cohort study showed that maternal exposure to any IPV during pregnancy and ever exposure to controlling behavior by the father of the child were independently associated with lower height-for-age mean Z-scores for children at around 40 to 46 months of age. However, after stratifying for the gender of the child these associations were significant only among girls.

### Limitations and strengths

One possible limitation of our research is that we measured ever exposure to controlling behavior by the father of the child instead of controlling behavior during pregnancy. With this approach is not possible to clearly differentiate whether the woman was exposed to these behaviors also during her current pregnancy. However, we agree with other authors that have recognized the continuous nature of men´s controlling behavior and its effects on women´s chronic disempowerment [26,27]. Thus, we believe that it is highly possible that these behaviors continued during and after pregnancy. Another possible limitation is that our cohort design does not allow us to adjust for maternal IPV exposure after pregnancy; which might also have influenced the child´s linear growth.

The external validity of our results was strengthened by the population-based design of this study. The cohort design allowed us to establish a temporal relationship between exposures and outcome variables. Selection bias is unlikely because no significant differences were found between the characteristics of the mothers of the children lost and found at follow-up. To reduce information bias field workers were extensively trained in how to collect IPV exposure data in privacy and in a non-judgmental manner. In order to minimize underreporting of IPV, data was collected twice during pregnancy. Efforts were made to ensure quality of anthropometric data.

Three hundred and seventy five children (178 boys and 197 girls) had height-for-age data available for analysis. This offered sufficient power to demonstrate a difference between exposure groups by approximately 0.3 SD score overall, and approximately 0.4 SD score among boys or girls.

### IPV and early child growth

In this study, ever controlling behavior by the father of the child was the most significant IPV exposure related to impaired child growth. Our findings are consistent with results from a recent cohort study in Bangladesh showing an association between maternal exposure to controlling behavior by the father of the child and lower height-for-age Z-scores in children aged 24 months [3].

Yount et al. have described several pathways by which IPV can impair child growth. IPV can affect maternal and fetal/infant health during and after pregnancy [12]. Our results showed that birthweight did not significantly modify the association between different forms of IPV during pregnancy and early child growth. This finding suggests that in our study, maternal exposure to one form of IPV (ever controlling behavior by the father of the child) influenced child growth in the postnatal rather than the prenatal period.

UNICEF has highlighted the paramount role that the care provided to a child has on his/her growth [28]. Controlling behavior by the father of the child can interfere with childcare in several ways. It can act by impairing women’s mental health, which has been shown to weaken women’s ability to fulfill their children’s needs and has also been associated with impaired child growth [28,29]. Some authors have suggested that this form of IPV might be more chronic and ongoing than other forms of IPV [26,27]. This might reflect on increased chronic rather than acute maternal stress.

In addition, controlling behavior might impair child linear growth by increasing the risk of lower respiratory tract infections and diarrheal diseases during the first year of life [4], which is in line with previous studies reporting that increased infectious illness frequency and severity are associated with poor growth [30,31].

Our results show that the gender of the child played a role in the association between ever maternal exposure to controlling behavior by the father of the child and linear growth. Bivariate analysis showed no gender differences in mean height-for-age Z-scores; however, with exposure to any IPV during pregnancy and ever controlling behavior by the father of the child, adjusted mean height-for-age Z-scores were significantly lower only among girls.

These results differ from previous population-based studies, which reported that girls experienced less chronic malnutrition than boys [32,33]. However, lower mean height-for-age Z-scores for girls might be explained by their increased exposure to inadequate care [34] and negative patterns of healthcare seeking behaviors [32,35,36], which could be aggravated by a mother’s lack of autonomy and control over family resources due to controlling behavior by her partner. Our results are in line with the findings from a cross-sectional study conducted in Nicaragua that found that young girls had higher odds of poor growth than boys [37].

In contrast, a study conducted in Bangladesh found that boys exposed to any form of VAW were more at risk of impaired growth than girls [3]. These differing findings might be related to different definitions of exposure between studies. The Bangladesh study used ever exposure to violence by any family member whereas we measured partner abuse during pregnancy.

## Conclusion

This study has contributed to the growing amount of evidence pointing to the pervasive effect of different forms of IPV on child health. Our study highlights the relevance of maternal autonomy for linear child growth, especially for young girls in the Nicaraguan context. Policy makers aiming to improve children’s nutritional status must develop interventions that include actions to identify and diminish mothers’ exposure to IPV.

## Abbreviations

ANCOVA, Analysis of covariance; CI, Confidence interval; HDSS, Health and demographic surveillance system; IPV, Intimate partner violence; IQR, Interquartile range; SD, Standard deviation; SRQ, Self-report questionnaire; VAW, Violence against women; WHO, World Health Organization.

## Competing interests

We authors declare that we do not have any competing interest.

## Authors’ contributions

MS and EV participated in the design of the study, as principal investigators, in recruiting women into the study, were responsible for the data collection, contributed to the analysis and helped to write the report. UH participated in the design of the study, as principal investigator, contributed to the analysis and helped to write the report. LÅP participated in the analysis and helped to write the report. All authors read and approved the final manuscript.

## Pre-publication history

The pre-publication history for this paper can be accessed here:

http://www.biomedcentral.com/1471-2431/12/82/prepub
